# Supplementary material for: Cystine-knot peptide inhibitors of HTRA1 bind to a cryptic pocket within the active site region
Source: Nat Commun. 2024 May 22;15:4359. doi: 10.1038/s41467-024-48655-w (PMC11111691; doi:10.1038/s41467-024-48655-w)
Supplement: Supplementary file 1 — Supplementary Information [file 41467_2024_48655_MOESM1_ESM.pdf]

# Supplementary Information

## Cystine-knot peptide inhibitors of HTRA1 bind to a cryptic pocket within the active site region

Yanjie Li[1*], Yuehua Wei[1*], Mark Ultsch[2], Wei Li[1], Wanjian Tang[1], Benjamin Tombling[1], Xinxin Gao[1], Yoana Dimitrova[2], Christian Gampe[3], Jakob Fuhrmann[1], Yingnan Zhang[1], Rami N. Hannoush[1#], Daniel Kirchhofer[1#]

[1]Department of Early Discovery Biochemistry, Genentech Inc., 1 DNA Way, South San Francisco, CA 94080

[2]Department of Structural Biology, Genentech Inc., 1 DNA Way, South San Francisco, CA 94080

[3]Department of Discovery Chemistry, Genentech Inc., 1 DNA Way, South San Francisco, CA 94080

*Equal contributions

# to whom correspondence should be addressed: dak@gene.com and ramihannoush@gmail.com

## Supplementary Figures

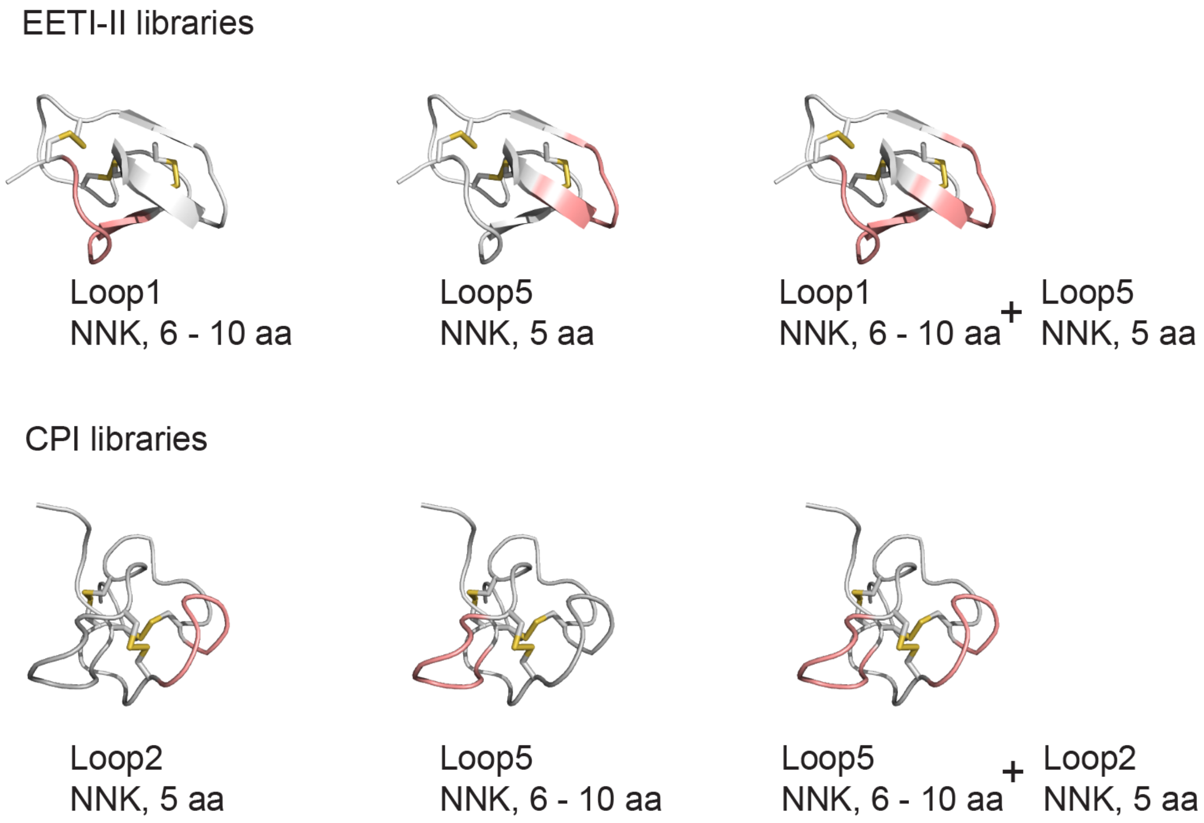


**Supplementary Figure 1. CKP phage library design.** The randomized regions are highlighted in salmon on the structures of EETI-II and CPI. NNK, randomization by NNK codon incorporation. aa, amino acid. EETI-II, *Ecballium elaterium* trypsin inhibitor II (PDB: 2ETI) [1]; CPI, carboxypeptidase A1 inhibitor (PDB: 4CPA) [2].

**a**

| EETI-II library | 1 | 2 | 3 | 4 | 5 | 6 | 7 | 8 | 9 | 10 | 11 | 12 | 13 | 14 | 15 | 16 | 17 | 18 | 19 | 20 | 21 | 22 | 23 | 24 | 25 | 26 | 27 | 28 | 29 | 30 | 31 | 32 |
|---|---|---|---|---|---|---|---|---|---|---|---|---|---|---|---|---|---|---|---|---|---|---|---|---|---|---|---|---|---|---|---|---|
| Peptide | | | L1 | | | | | | | | | | | L2 | | | | | | L3 | | | | L4 | | L5 | | | | | | |
| EETI-II | G | C | P | R | I | L | M | R | - | - | - | - | C | K | Q | D | S | D | C | L | A | G | C | V | C | G | P | N | G | F | C | G |
| 1G10 | G | C | I | Q | M | W | E | H | - | - | - | - | C | K | Q | D | S | D | C | L | A | G | C | V | C | L | M | F | N | Q | C | G |
| 1C2 | G | C | G | R | W | K | L | Q | N | F | V | - | C | K | Q | D | S | D | C | L | A | G | C | V | C | G | P | N | G | F | C | G |
| 1D5 | G | C | T | G | K | H | G | V | Y | W | F | S | C | K | Q | D | S | D | C | L | A | G | C | V | C | G | P | N | G | F | C | G |
| 1A11 | G | C | G | R | W | K | I | S | W | R | - | - | C | K | Q | D | S | D | C | L | A | G | C | V | C | G | P | N | G | F | C | G |
| 1C10 | G | C | Q | G | K | Y | G | V | F | W | F | A | C | K | Q | D | S | D | C | L | A | G | C | V | C | G | P | N | G | F | C | G |
| 1C7 | G | C | M | W | F | W | E | N | D | W | L | L | C | K | Q | D | S | D | C | L | A | G | C | V | C | S | M | F | G | E | C | G |
| 1G2 | G | C | R | S | Q | K | F | R | F | T | - | - | C | K | Q | D | S | D | C | L | A | G | C | V | C | G | P | N | G | F | C | G |
| 1H7 | G | C | H | E | L | W | E | W | - | - | - | - | C | K | Q | D | S | D | C | L | A | G | C | V | C | L | M | Y | E | K | C | D |
| 1A12 | G | C | K | G | R | Y | G | S | Y | W | M | T | C | K | Q | D | S | D | C | L | A | G | C | V | C | G | P | N | G | F | C | G |
| 2A3-P1 | G | C | K | R | W | R | V | W | - | - | - | - | C | K | Q | D | S | D | C | L | A | G | C | V | C | E | G | A | Q | W | C | G |

**b**

| CPI library | 1 | 2 | 3 | 4 | 5 | 6 | 7 | 8 | 9 | 10 | 11 | 12 | 13 | 14 | 15 | 16 | 17 | 18 | 19 | 20 | 21 | 22 | 23 | 24 | 25 | 26 | 27 | 28 | 29 | 30 | 31 | 32 | 33 | 34 | 35 | 36 | 37 |
|---|---|---|---|---|---|---|---|---|---|---|---|---|---|---|---|---|---|---|---|---|---|---|---|---|---|---|---|---|---|---|---|---|---|---|---|---|---|
| Peptide | N terminus | | | | | | L1 | | | | L2 | | | | | | L3 | | | | | | L4 | | | L5 | | | | | | | C terminus | | | | |
| CPI | H | A | D | P | I | C | N | K | P | C | K | T | H | D | D | C | S | G | A | W | F | C | Q | A | C | W | N | S | A | R | T | C | G | P | Y | V | G |
| 3A7 | H | A | D | P | I | C | N | K | P | C | K | T | H | D | D | C | S | G | A | W | F | C | Q | A | C | Y | F | A | N | W | R | C | G | P | Y | V | G |
| 3B3 | H | A | D | P | I | C | N | K | P | C | K | T | H | D | D | C | S | G | A | W | F | C | Q | A | C | Y | Y | A | T | W | S | C | G | P | Y | V | G |

**c**

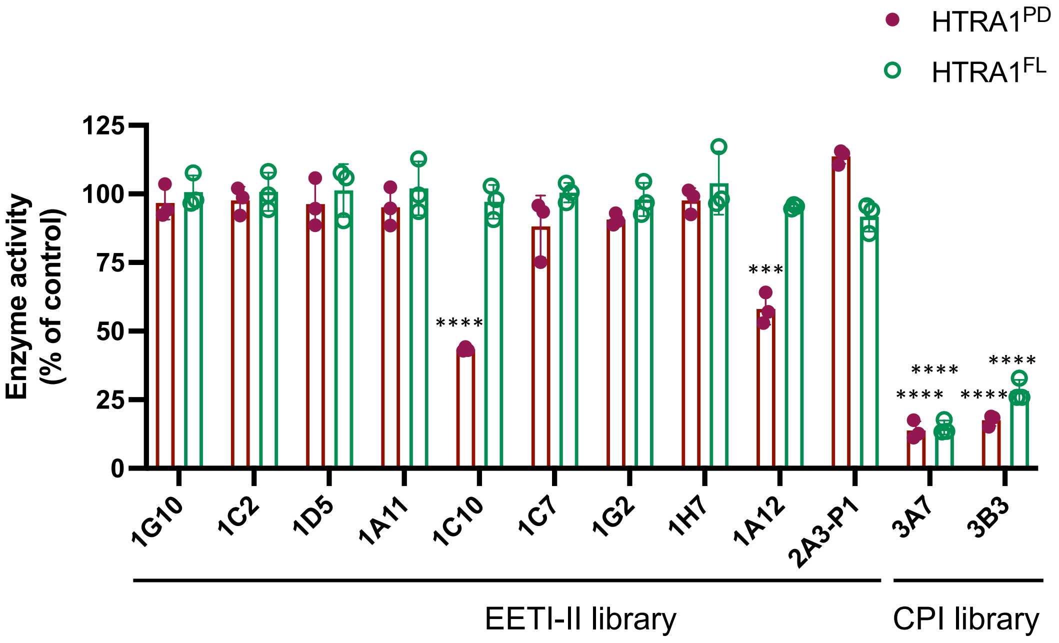


**Supplementary Figure 2. Sequences of HTRA1-binding CKPs and identification of inhibitors in enzymatic assays. a and b.** Sequences of the synthesized hits from screening of EETI-II libraries (a) and CPI libraries (b); the CKP loops are individually colored and changed

residues compared to the original scaffold sequences (top row) are in red. **c.** Synthesized CKPs (from a and b) were tested for HTRA1 inhibition in the fluorescence-quenched casein-BODIPY® assay using 30 nM $HTRA1^{FL}$ or 30 nM $HTRA1^{PD}$ and 2 μM CKP. The values are the mean ± S.D. of three independent experiments. ***, $P = 0.0002$; ****, $P < 0.0001$; two-sided, unpaired Student's *t* test comparing CKPs with DMSO controls.

**a**

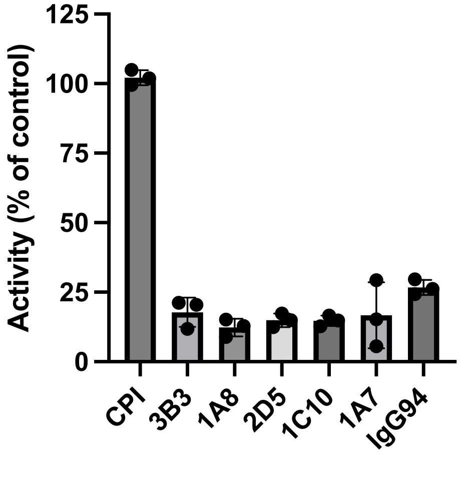


**b**

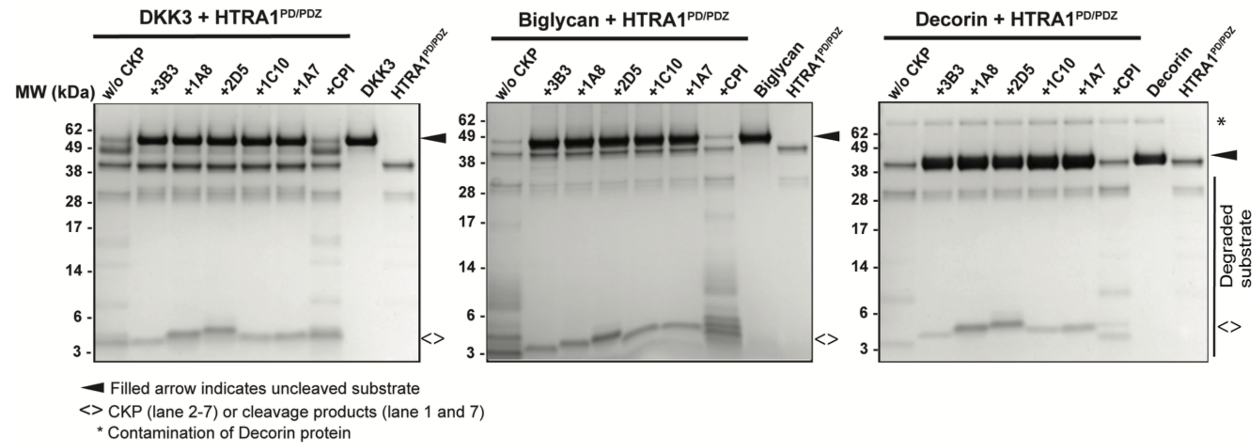


**Supplementary Figure 3. CKPs inhibit HTRA1$^{PD/PDZ}$ and the endogenous melanoma cell-derived HTRA1. a.** Conditioned medium of C32 melanoma cells containing endogenous full-length HTRA1 was incubated with the CKPs of the X5 and X8 group (final concentration of 2 μM) or the anti-HTRA1 antibody IgG94 (final concentration of 0.5 μM; positive control) for 60 min at 37°C and activities were measured after addition of the synthetic substrate H2-OPT. The values are the mean ± S.D. of three independent experiments. HTRA1 was significantly inhibited by the 3B3-derived CKPs and IgG94 (P<0.001), but not by CPI; two-sided, unpaired Student's *t* test comparing CKPs/IgG94 with DMSO controls. **b.** The parent 3B3 and affinity-matured CKPs of the X5 and X8 group completely inhibited HTRA1$^{PD/PDZ}$-mediated cleavage of

the macromolecular substrates DKK3, biglycan and decorin, while the CPI scaffold had no effect. The CKP bands (lanes 2-7) in some cases overlap with a substrate degradation product. Values in **a** show the mean ± S.D. of at least three independent experiments. Images in **b** are representative of two independent experiments.

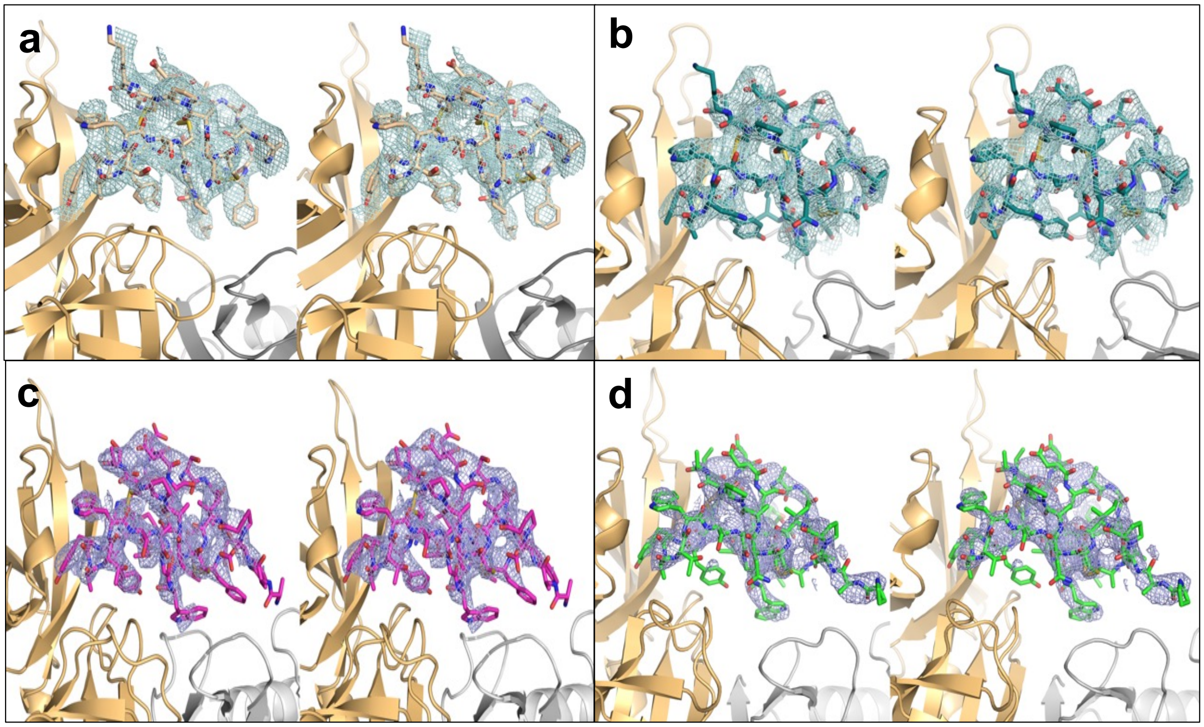


**Supplementary Figure 4. Electron density 2mFo-DFc omit maps (in stereo) contoured at 1 times rmsd shown for all CKP atoms. a.** CKP-3B3 (brown) bound to HTRA1 chain A (brown) and neighboring protomer chain C (grey). **b.** CKP-3A7 (teal) bound to HTRA1 chain A (brown) and neighboring protomer chain C (grey). **c.** CKP-1A8 (magenta) bound to HTRA1 chain B (brown) and neighboring protomer chain A (grey). **d.** CKP-1G10 (green) bound to HTRA1 chain C (brown) and neighboring protomer chain B (grey).

C6 – C22 | C10 – C25 | C16 – C32

**a**

| | 1 | 2 | 3 | 4 | 5 | 6 | 7 | 8 | 9 | 10 | 11 | 12 | 13 | 14 | 15 | 16 | 17 | 18 | 19 | 20 | 21 | 22 | 23 | 24 | 25 | 26 | 27 | 28 | 29 | 30 | 31 | 32 | 33 | 34 | 35 | 36 | 37 |
|---|---|---|---|---|---|---|---|---|---|---|---|---|---|---|---|---|---|---|---|---|---|---|---|---|---|---|---|---|---|---|---|---|---|---|---|---|---|
| Peptide | N terminus | | | | | | L1 | | | | L2 | | | | | | L3 | | | | | | L4 | | | L5 | | | | | | | C terminus | | | | |
| CPI | H | A | D | P | I | C | N | K | P | C | K | T | H | D | D | C | S | G | A | W | F | C | Q | A | C | W | N | S | A | R | T | C | G | P | Y | V | G |
| 3B3 | H | A | D | P | I | C | N | K | P | C | K | T | H | D | D | C | S | G | A | W | F | C | Q | A | C | Y | Y | A | T | W | S | C | G | P | Y | V | G |

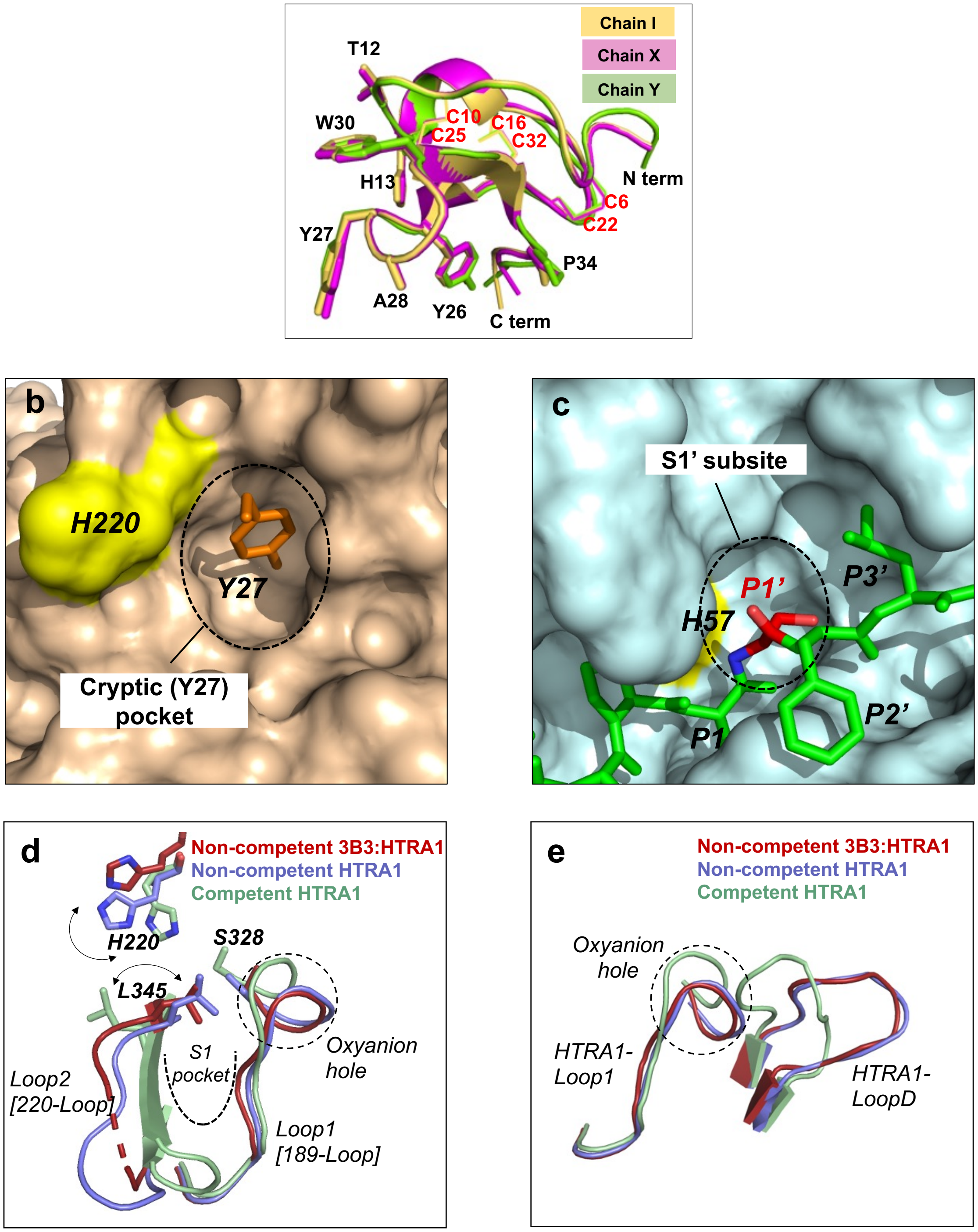


**Supplementary Figure 5. Location of the cryptic pocket in the 3B3:HTRA1$^{PD(SA)}$ complex and the non-competent HTRA1 active site. a.** Sequences and disulfide bond network of 3B3

compared to the parent CPI scaffold and superposition of 3B3 chain I (yellow), chain X (magenta) and chain Y (green) as cartoons, with HTRA1 binding residues as sticks; cysteines forming the three disulfide bonds are indicated. **b.** Surface rendering of HTRA1 (brown) with Y27 of 3B3 (orange sticks) inserted into the cryptic pocket (dotted circle), which is in close proximity to the S1’ subsite in thrombin. **c.** Surface rendering of thrombin (light blue) with the P1’-serine residue (red sticks) of the bound PAR-1 substrate peptide (PDB: 3LU9) inserted into the S1’ pocket. Catalytic histidine in b and c is in yellow. **d.** Cartoon representation of the non-competent active sites of 3B3:HTRA1$^{PD(SA)}$ (red) and apo-HTRA1 (PDB: 3TJN-chain A, blue) vs the competent conformation of apo-HTRA1 (PDB: 3TJN-chain B, green). Indicated are the flipped side chains of H220 and L345, the S1 pocket and the oxyanion hole. **e.** The non-competent conformations of the active site Loop1 [189-Loop] and LoopD [148-Loop] in 3B3:HTRA1 (red) and non-competent apo-HTRA1 (PDB: 3TJN-chain A; blue) compared to the competent conformation of apo-HTRA1 (PDB: 3TJN-chain B; green).

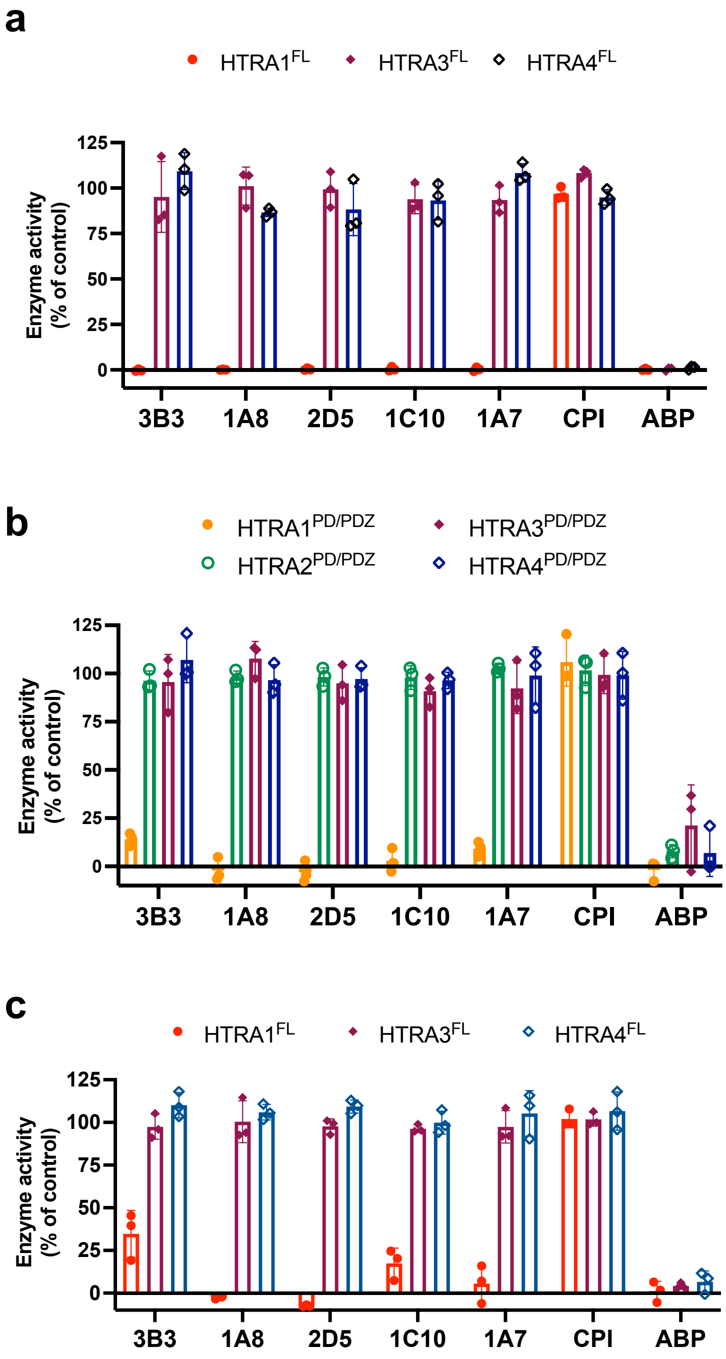


**Supplementary Figure 6. Selectivity of CKPs in enzyme assays with the synthetic substrate H2-OPT and in casein cleavage assays. a.** Synthetic substrate (H2-OPT) assay with full length forms of HTRA1,3,4 ($HTRA1^{FL}$, $HTRA3^{FL}$, $HTRA4^{FL}$). At 2 μM concentration the parent 3B3 and affinity-matured CKPs (X5 and X8 group) only inhibited $HTRA1^{FL}$, but not $HTRA3^{FL}$ or $HTRA4^{FL}$. The specific activities of the enzymes and enzyme:inhibitor ratios were

as follows: HTRA1$^{FL}$, 39178 mRFU/min/nM – 1:2000; HTRA3$^{FL}$, 11581 mRFU/min/nM – 1:667; HTRA4$^{FL}$, 7134 mRFU/min/nM – 1:333. Values are the mean ± S.D. for at least three independent experiments. For HTRA3$^{FL}$ and HTRA4$^{FL}$ there was no statistically significant reduction in activity by the CKPs vs DMSO controls, except for HTRA4$^{FL}$ by 1A8 (13% reduction; $p<0.001$) (two-sided, unpaired Student's *t* test). HTRA1$^{FL}$ was significantly inhibited by the CKPs ($P<0.0001$) except CPI. All enzymes were significantly inhibited by 7mer activity-based probe DPMFKLV-phosphonate (ABP) ($P<0.0001$). **b.** Casein cleavage assay (fluorescence-quenched casein-BODIPY® substrate) with HTRA1-4$^{PD/PDZ}$ and 2 μM CKPs. The specific activities of the enzymes and enzyme:inhibitor ratios were as follows: HTRA1$^{PD/PDZ}$, 104 mRFU/min/nM – 1:67; HTRA2$^{PD/PDZ}$, 33 mRFU/min/nM – 1:67; HTRA3$^{PD/PDZ}$, 0.5 mRFU/min/nM – 1:20. HTRA4$^{PD/PDZ}$, 0.3 mRFU/min/nM – 1:6.7. For HTRA2-4$^{PD/PDZ}$ there was no statistically significant reduction in activity by the CKPs vs DMSO controls (two-sided, unpaired Student's *t* test). HTRA1$^{PD/PDZ}$ was significantly inhibited by the CKPs ($P<0.001$) except CPI. All enzymes were significantly inhibited the ABP ($P<0.01$). **c.** Casein cleavage assay with full length forms of HTRA1,3,4 (HTRA1$^{FL}$, HTRA3$^{FL}$, HTRA4$^{FL}$) and 2 μM CKPs. The specific activities of the enzymes and enzyme:inhibitor ratios were as follows: HTRA1$^{FL}$, 168 mRFU/min/nM – 1:67; HTRA3$^{FL}$, 2.3 mRFU/min/nM – 1:67; HTRA4$^{FL}$, 1.9 mRFU/min/nM – 1:67. For HTRA3$^{FL}$ and HTRA4$^{FL}$ there was no statistically significant reduction in activity by the CKPs vs DMSO controls (two-sided, unpaired Student's *t* test). HTRA1$^{FL}$ was significantly inhibited by the CKPs ($P<0.01$) except CPI. All enzymes were significantly inhibited the ABP ($P<0.0001$). The values in Fig. S6a-c are the mean ± S.D. of at least three independent experiments.

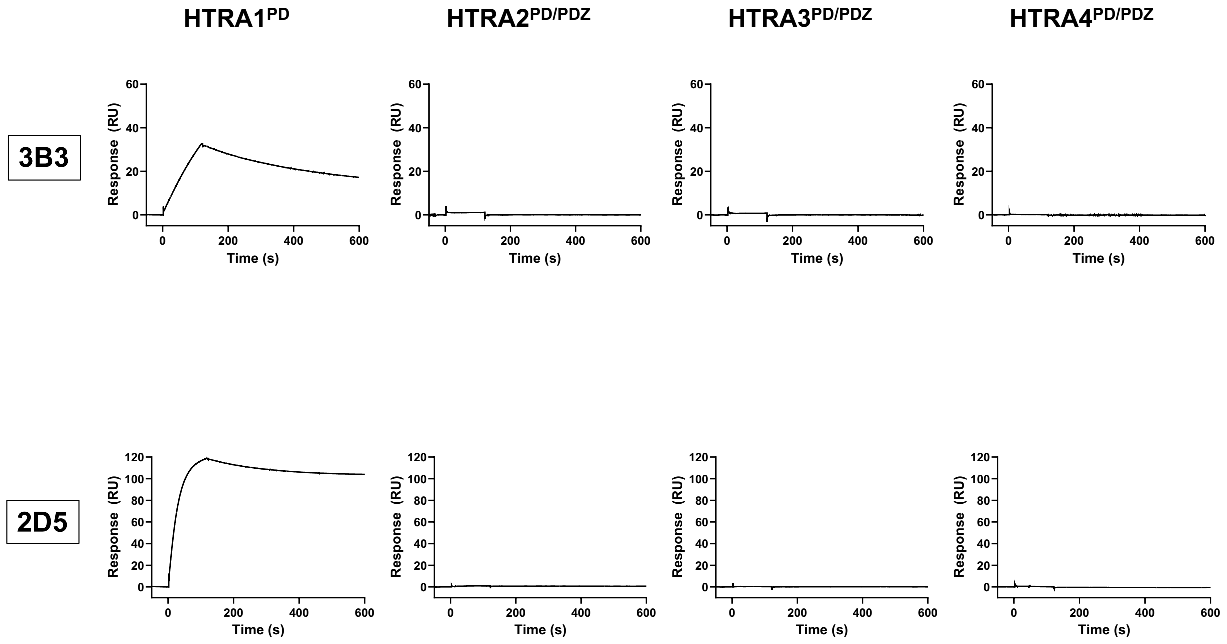


**Supplementary Figure 7. SPR sensorgrams showing kinetics of CKP binding to HTRA family proteases.** HTRA proteins were immobilized on CM5 sensor chips via their His-tags and 3B3 and 2D5 were both tested at 1 µM. All sensorgrams are representative of three independent experiments.

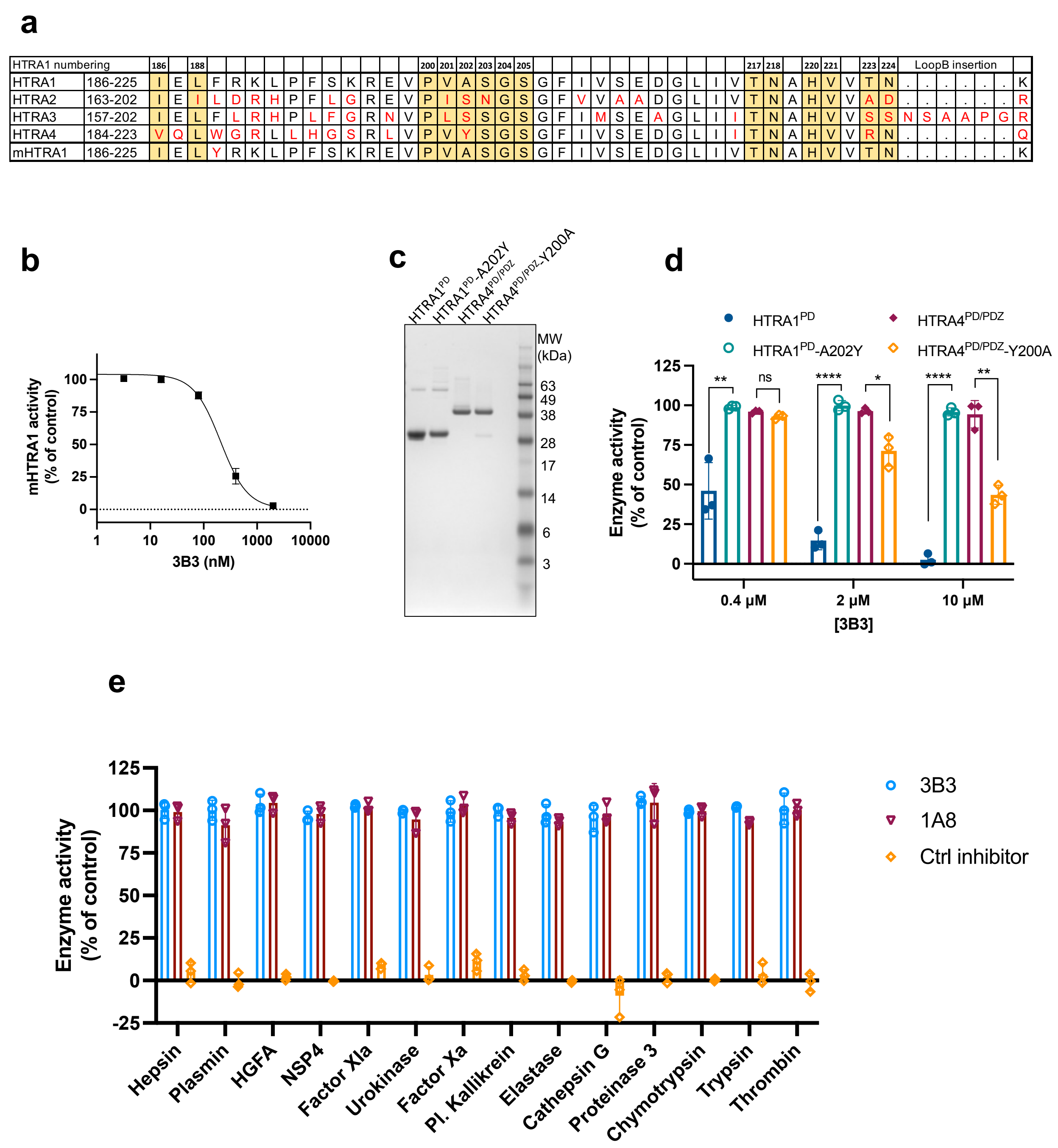


**Supplementary Figure 8. The HTRA1 cryptic pocket determines CKP selectivity. a.** Sequence alignment of HTRA proteases comprising the region of the HTRA1 cryptic pocket. Residue changes in HTRA2-4 and murine HTRA1 (mHTRA1) in comparison to HTRA1 are shown in red. HTRA1 residues contacting 3B3 are highlighted in yellow. **b.** mHTRA1 has no sequence changes in the pocket binding region (a) and HTRA1$^{PD}$ is inhibited by 3B3 with an $IC_{50}$ of 209 ± 32 nM (mean ± S.D. of three independent experiments). **c.** SDS-PAGE (reducing

conditions) of wildtype HTRA proteins (HTRA1$^{PD}$, HTRA4$^{PD/PDZ}$) and the chimeric proteins HTRA1$^{PD}$-A202Y and HTRA4$^{PD/PDZ}$-Y200A. **d.** Inhibition of wildtype and chimeric proteases by 3B3. The wildtype form of HTRA4 (HTRA4$^{PD/PDZ}$) is completely resistant to 3B3, but becomes susceptible to inhibition when the Y200 residue is replaced with the corresponding alanine residue of HTRA1 (Y200A). HTRA4$^{PD/PDZ}$-Y200A is inhibited by 57% at 10 µM 3B3. Conversely, 3B3 no longer inhibits the chimeric HTRA1$^{PD}$-A202Y, in which the HTRA1-A202 residue is replaced with the corresponding tyrosine residue of HTRA4. The specific activities of the enzymes and enzyme:inhibitor ratios for 0.4µM/2µM/10µM 3B3 were as follows: HTRA1$^{PD}$, 66590 mRFU/min/nM – 1:400/1:2000/1:10000; HTRA1$^{PD}$-A202Y, 2516 mRFU/min/nM – 1:16/1:80/1:400; HTRA4$^{PD/PDZ}$, 40 mRFU/min/nM – 1:0.4/1:2/1:10; HTRA4$^{PD/PDZ}$-Y200A, 42 mRFU/min/nM – 1:0.4/1:2/1:10. *, $P = 0.0116$; **, $P = 0.0068$ (0.4 µM, HTRA1$^{PD}$ vs HTRA1$^{PD}$-A202Y), $P = 0.0011$ (10 µM, HTRA4$^{PD/PDZ}$ vs HTRA4$^{PD/PDZ}$-Y200A); ****, $P < 0.0001$; ns, not significant; two-sided, unpaired Student's *t* test comparing wildtype with corresponding mutant protease as indicated. **e.** 3B3 and 1A8 (at a concentration of 2 µM) do not inhibit the enzymatic activities of trypsin fold serine proteases of the S1A subfamily (same protease panel as shown in Fig. 4c). Control inhibitors were PMSF or leupeptin, except for NSP4 (PK401 activity-based probe) [3], FXIa and urokinase (PAI-1). The enzyme:inhibitor ratios ranged from 1:25 (elastase) to 1:8000 (plasma kallikrein). The values in **b**, **d** and **e** are the mean ± S.D. of at least three independent experiments.

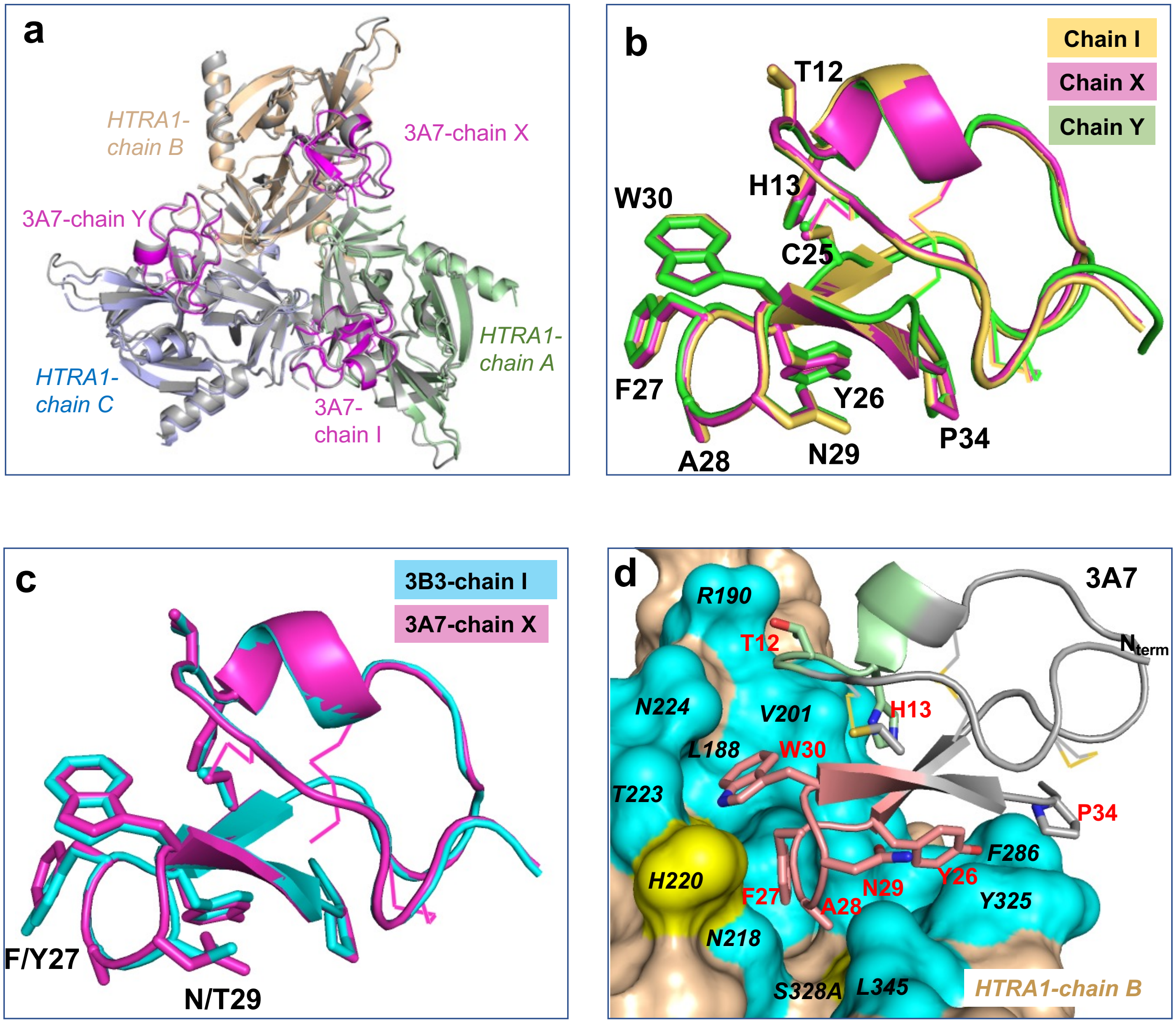


**Supplementary Figure 9. The 3A7:HTRA1$^{PD(SA)}$ complex. a.** Superposition of the 3B3:HTRA1$^{PD(SA)}$ complex (gray cartoon) with the 3A7:HTRA1$^{PD(SA)}$ complex with the three HTRA1$^{PD(SA)}$ chains in different colors and the three 3A7 copies in magenta. **b.** Superposition of the three 3A7 chains with contact residues as sticks. Similar to the 3B3 structure the two (chain Y) or three (chain X, I) N-terminal residues were not resolved and neither were the two C-terminal residues V36 and G37. **c.** Superposition of the 3A7 reference chain X (magenta) and 3B3 reference chain I (cyan). Two of the three residues that differ between 3A7 and 3B3 are labeled; the third residue, R31, is a non-contact residue and its side chain was not resolved in chain X. **d.** HTRA1-chain B is shown as surface representation (brown) and HTRA1 residues within 4 Å of 3A7 (chain X) are in cyan (catalytic H220 and S328A in yellow) and labeled in

black italics. The important contact Loops 2 and 5 of 3A7 are in green and salmon, respectively and contact residues are shown as sticks and labeled in red.

**a**

| Peptide | IC50 ± S.D. (nM) | 1 | 2 | 3 | 4 | 5 | 6 | 7 | 8 | 9 | 10 | 11 | 12 | 13 | 14 | 15 | 16 | 17 | 18 | 19 | 20 | 21 | 22 | 23 | 24 | 25 | 26 | 27 | 28 | 29 | 30 | 31 | 32 | 33 | 34 | 35 | 36 | 37 | 38 | 39 | 40 |
|---|---|---|---|---|---|---|---|---|---|---|---|---|---|---|---|---|---|---|---|---|---|---|---|---|---|---|---|---|---|---|---|---|---|---|---|---|---|---|---|---|---|
| | | N terminus | | | | | | L1 | | | | L2 | | | | | | L3 | | | | | | L4 | | | L5 | | | | | | | C terminus | | | | | | | |
| 1A8 | 1.9 ± 0.2 | H | A | D | P | I | C | N | K | P | C | K | T | H | D | D | C | S | G | A | W | F | C | Q | T | C | Y | Y | A | T | W | S | C | G | W | G | L | R | Q | I | D |
| 1A8.K11E | 1.0 ± 0.3 | H | A | D | P | I | C | N | K | P | C | E | T | H | D | D | C | S | G | A | W | F | C | Q | T | C | Y | Y | A | T | W | S | C | G | W | G | L | R | Q | I | D |
| 1A8.K11D | 1.2 ± 0.4 | H | A | D | P | I | C | N | K | P | C | D | T | H | D | D | C | S | G | A | W | F | C | Q | T | C | Y | Y | A | T | W | S | C | G | W | G | L | R | Q | I | D |
| 1A8.D14DaMe | 5.0 ± 0.3 | H | A | D | P | I | C | N | K | P | C | K | T | H | DaMe | D | C | S | G | A | W | F | C | Q | T | C | Y | Y | A | T | W | S | C | G | W | G | L | R | Q | I | D |
| 1A8.Y26Y(3,5diF) | 10.6 ± 1.3 | H | A | D | P | I | C | N | K | P | C | K | T | H | D | D | C | S | G | A | W | F | C | Q | T | C | Y(3,5diF) | Y | A | T | W | S | C | G | W | G | L | R | Q | I | D |
| 1A8.Y26Y(3Cl) | 2.4 ± 0.3 | H | A | D | P | I | C | N | K | P | C | K | T | H | D | D | C | S | G | A | W | F | C | Q | T | C | Y(3Cl) | Y | A | T | W | S | C | G | W | G | L | R | Q | I | D |
| 1A8.Y27F(4Cl) | 27.7 ± 4.2 | H | A | D | P | I | C | N | K | P | C | K | T | H | D | D | C | S | G | A | W | F | C | Q | T | C | Y | F(4Cl) | A | T | W | S | C | G | W | G | L | R | Q | I | D |
| 1A8.Y27F(4F) | 6.8 ± 0.6 | H | A | D | P | I | C | N | K | P | C | K | T | H | D | D | C | S | G | A | W | F | C | Q | T | C | Y | F(4F) | A | T | W | S | C | G | W | G | L | R | Q | I | D |
| 1A8.Y27F(3,4diF) | 24.6 ± 2.8 | H | A | D | P | I | C | N | K | P | C | K | T | H | D | D | C | S | G | A | W | F | C | Q | T | C | Y | F(3,4diF) | A | T | W | S | C | G | W | G | L | R | Q | I | D |
| 1A8.Y27F(4Me) | 15.7 ± 1.8 | H | A | D | P | I | C | N | K | P | C | K | T | H | D | D | C | S | G | A | W | F | C | Q | T | C | Y | F(4Me) | A | T | W | S | C | G | W | G | L | R | Q | I | D |
| 1A8.Y27hF | 265.8 ± 46.8 | H | A | D | P | I | C | N | K | P | C | K | T | H | D | D | C | S | G | A | W | F | C | Q | T | C | Y | hF | A | T | W | S | C | G | W | G | L | R | Q | I | D |
| 1A8.Y27Y(3Cl) | 7.8 ± 0.2 | H | A | D | P | I | C | N | K | P | C | K | T | H | D | D | C | S | G | A | W | F | C | Q | T | C | Y | Y(3Cl) | A | T | W | S | C | G | W | G | L | R | Q | I | D |
| 1A8.Y27(3,5diCl) | 33.8 ± 4.3 | H | A | D | P | I | C | N | K | P | C | K | T | H | D | D | C | S | G | A | W | F | C | Q | T | C | Y | Y(3,5diCl) | A | T | W | S | C | G | W | G | L | R | Q | I | D |
| ***Loop1 library:*** | | | | | | | | | | | | | | | | | | | | | | | | | | | | | | | | | | | | | | | | | |
| 1A8.L1.1 | 2.3 ± 0.1 | H | A | D | P | I | C | G | S | Q | C | K | T | H | D | D | C | S | G | A | W | F | C | Q | T | C | Y | Y | A | T | W | S | C | G | W | G | L | R | Q | I | D |
| 1A8.L1.2 | 3.2 ± 0.1 | H | A | D | P | I | C | G | H | Q | C | K | T | H | D | D | C | S | G | A | W | F | C | Q | T | C | Y | Y | A | T | W | S | C | G | W | G | L | R | Q | I | D |
| ***Loop5 library:*** | | | | | | | | | | | | | | | | | | | | | | | | | | | | | | | | | | | | | | | | | |
| 1A8.L5.1 | 1.0 ± 0.1 | H | A | D | P | I | C | N | K | P | C | K | T | H | D | D | C | S | G | A | W | F | C | Q | T | C | Y | Y | A | N | W | S | C | G | W | G | L | R | Q | I | D |
| 1A8.L5.2 | 2.0 ± 0.2 | H | A | D | P | I | C | N | K | P | C | K | T | H | D | D | C | S | G | A | W | F | C | Q | T | C | Y | Y | A | S | W | S | C | G | W | G | L | R | Q | I | D |

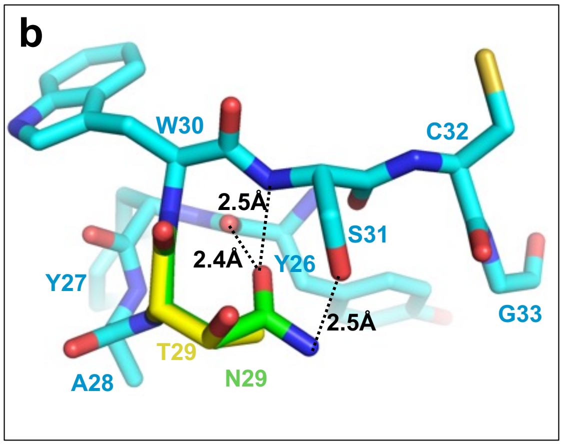


**Supplementary Figure 10. Sequences and activities of CKPs obtained from structure-based designs and from affinity maturations of 3B3-Loop1 and Loop5. a.** $IC_{50}$ values of synthesized CKPs based on structure-based designs and from screening libraries with diversity in 3B3-Loop1 and in Loop5. The amino acid changes of the obtained four hits from Loop1 and 5 libraries were incorporated into 1A8 and synthesized. The values are the mean ± S.D. of at least three independent experiments. Changed residues in comparison to the parent 1A8 (top row) are in red. Unnatural amino acids incorporated into Loop2 and Loop5 are: DaMe, Cα-methyl-aspartate; Y(3,5diF), 3,5-di-fluoro-tyrosine; Y(3Cl), 3-chloro-tyrosine; F(4Cl), 4-chloro-phenylalanine; F(4F), 4-fluoro-phenylalanine; F(3,4diF), 3,4-di-fluoro-phenylalanine; F(4Me), 4-methyl-phenylalanine; hF, homo-phenylalanine; Y(3Cl), 3-chloro-tyrosine; Y(3,5diCl), 3,5-di-chloro-tyrosine. **b.** The N29 residue from the Loop5 affinity maturation modelled in the 3B3 structure (chain I) is shown in green. Compared to the wildtype T29 residue (yellow), the N29

forms H-bonds with sidechain hydroxyl and backbone amine of S31 and backbone carbonyl of Y26.

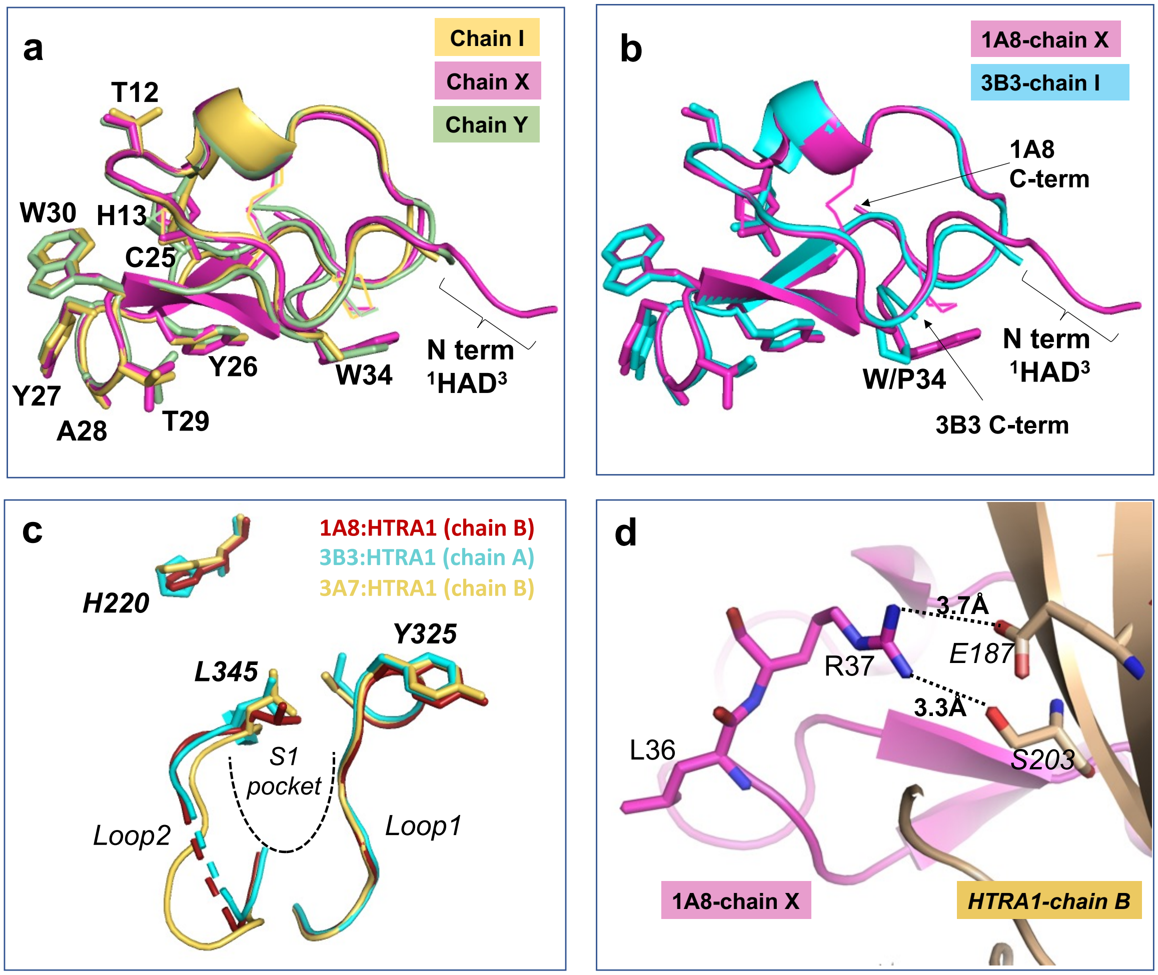


**Supplementary Figure 11. The 1A8:HTRA1$^{PD(SA)}$ complex. a.** Superposition of the three 1A8 chains in the HTRA1$^{PD(SA)}$ trimer are shown in different colors and side chains of contact residues as sticks. Chain X has a fully resolved N terminus, which includes the three residues H1, A2 and D3, whereas chain I and chain Y are missing H1-D3 and H1-A2, respectively. **b.** Superposition of the reference 1A8-chain X (magenta) and the reference 3B3-chain I (cyan) with contact residues shown as sticks. The changed C-terminal residue W34 (P34 in 3B3) is labeled. The resolved N-terminal residues $^{1}$HAD$^{3}$ of 1A8-chain X are missing in 3B3-chain I. **c.** Comparison of the non-competent active site conformation (cartoon) of the 1A8:HTRA1 complex (red) with that of 3B3:HTRA1 (cyan) and of 3A7:HTRA1 (yellow). The catalytic H220 and residues L345 and Y325 are shown as sticks. **d.** Interaction of the guanidinium group of the C-terminal 1A8 residue R37 (there was no electron density for residues Q38, I39 and D40) with E187 and S203 of HTRA1 (chain B; brown).

**a**

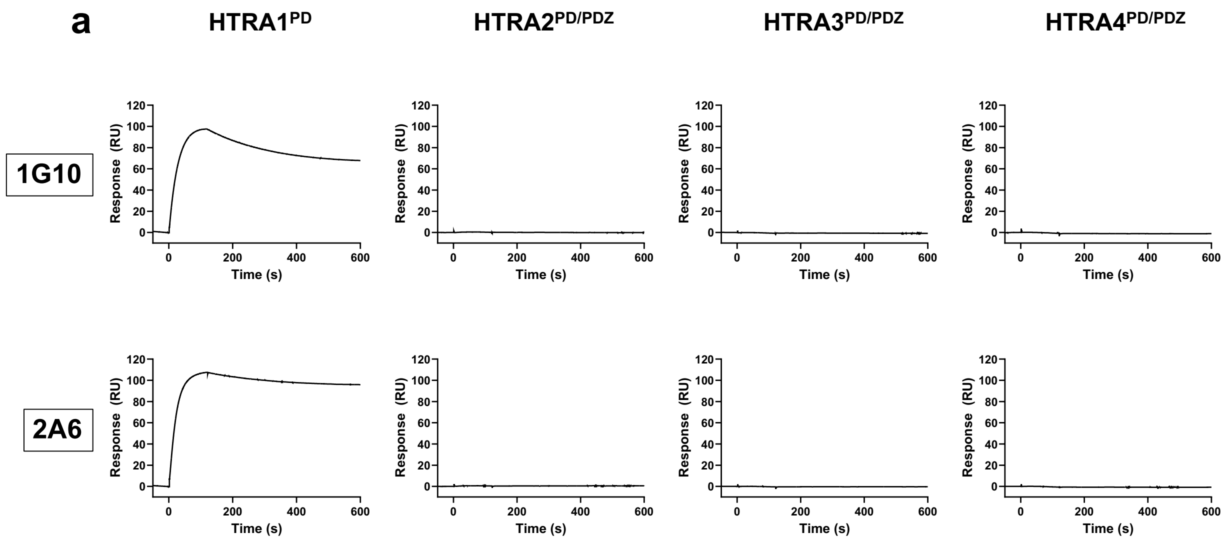


**b**

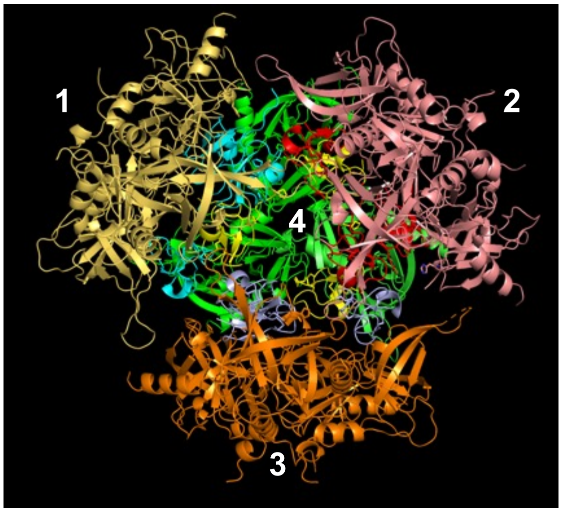


**c**

| | | -1 | 1 | 2 | 3 | 4 | 5 | 6 | 7 | 8 | 9 | 10 | 11 | 12 | 13 | 14 | 15 | 16 | 17 | 18 | 19 | 20 | 21 | 22 | 23 | 24 | 25 | 26 | 27 | 28 | 29 | 30 | 31 | 32 | 33 | 34 | 35 | 36 | 37 | 38 | 39 | 40 |
|---|---|---|---|---|---|---|---|---|---|---|---|---|---|---|---|---|---|---|---|---|---|---|---|---|---|---|---|---|---|---|---|---|---|---|---|---|---|---|---|---|---|---|
| **Peptide** | **IC50 ± S.D. (nM)** | **N terminus** | | | | | | | **L1** | | | | **L2** | | | | | | **L3** | | | | | | **L4** | | | **L5** | | | | | | | **C terminus** | | | | | | | |
| 1A8 | 1.90 ± 0.15 | | H | A | D | P | I | C | N | K | P | C | K | T | H | D | D | C | S | G | A | W | F | C | Q | T | C | Y | Y | A | T | W | S | C | G | W | G | L | R | Q | I | D |
| 1D3 | 0.87 ± 0.23 | I | W | P | D | P | I | C | N | K | P | C | K | T | H | D | D | C | S | G | A | W | F | C | Q | T | C | Y | Y | A | T | W | S | C | G | W | G | L | R | Q | I | D |
| 1G10 | 0.66 ± 0.12 | Y | P | V | D | P | I | C | N | K | P | C | K | T | H | D | D | C | S | G | A | W | F | C | Q | T | C | Y | Y | A | T | W | S | C | G | W | G | L | R | Q | I | D |
| ***N-term, L5 combo*** | | | | | | | | | | | | | | | | | | | | | | | | | | | | | | | | | | | | | | | | | | |
| 1D3-N | 0.63 ± 0.02 | I | W | P | D | P | I | C | N | K | P | C | K | T | H | D | D | C | S | G | A | W | F | C | Q | T | C | Y | Y | A | N | W | S | C | G | W | G | L | R | Q | I | D |
| 1G10-N | 0.96 ± 0.25 | Y | P | V | D | P | I | C | N | K | P | C | K | T | H | D | D | C | S | G | A | W | F | C | Q | T | C | Y | Y | A | N | W | S | C | G | W | G | L | R | Q | I | D |
| ***N-term, C-term combo*** | | | | | | | | | | | | | | | | | | | | | | | | | | | | | | | | | | | | | | | | | | |
| 1D3-I | 0.52 ± 0.09 | I | W | P | D | P | I | C | N | K | P | C | K | T | H | D | D | C | S | G | A | W | F | C | Q | T | C | Y | Y | A | T | W | S | C | G | W | G | I | R | | | |
| 1G10-I | 0.65 ± 0.12 | Y | P | V | D | P | I | C | N | K | P | C | K | T | H | D | D | C | S | G | A | W | F | C | Q | T | C | Y | Y | A | T | W | S | C | G | W | G | I | R | | | |
| ***N-term, L5, C-term combo*** | | | | | | | | | | | | | | | | | | | | | | | | | | | | | | | | | | | | | | | | | | |
| 1D3-NI | 0.59 ± 0.09 | I | W | P | D | P | I | C | N | K | P | C | K | T | H | D | D | C | S | G | A | W | F | C | Q | T | C | Y | Y | A | N | W | S | C | G | W | G | I | R | | | |
| 1G10-NI | 0.60 ± 0.06 | Y | P | V | D | P | I | C | N | K | P | C | K | T | H | D | D | C | S | G | A | W | F | C | Q | T | C | Y | Y | A | N | W | S | C | G | W | G | I | R | | | |
| ***N-term, L2, L5, C-term combo*** | | | | | | | | | | | | | | | | | | | | | | | | | | | | | | | | | | | | | | | | | | |
| 1D3-ENI | 0.27 ± 0.02 | I | W | P | D | P | I | C | N | K | P | C | E | T | H | D | D | C | S | G | A | W | F | C | Q | T | C | Y | Y | A | N | W | S | C | G | W | G | I | R | | | |
| 1G10-ENI | 0.30 ± 0.05 | Y | P | V | D | P | I | C | N | K | P | C | E | T | H | D | D | C | S | G | A | W | F | C | Q | T | C | Y | Y | A | N | W | S | C | G | W | G | I | R | | | |

**Supplementary Figure 12. Selectivity of CKPs derived from N- and C-terminal extension libraries, crystal packing of the 1G10:HTRA1$^{PD(SA)}$ complex, and inhibitory activities of combination CKPs. a.** HTRA proteins were immobilized on CM5 sensor chips via their His-tags and the inhibitory CKPs from the N- and C-terminal extension libraries, 1G10 and 2A6 respectively, were tested at 1 μM. The sensorgrams are representative of three independent experiments. **b.** Crystal packing of the 1G10:HTRA1$^{PD(SA)}$ complexes, which form a cage-like structure containing four trimers (numbered 1-4). HTRA1$^{PD(SA)}$ trimers are colored in dark yellow, salmon, orange and green; the bound 1G10 CKPs are in cyan, red, light blue and yellow. **c.** Sequences and $IC_{50}$ values of 1D3- and 1G10-derived CKPs combining optimal residue changes in Loop2 (E11, "E"), Loop5 (N29, "N") and C terminus (I36, "I"). The values are the mean ± S.D. of at least three independent experiments.

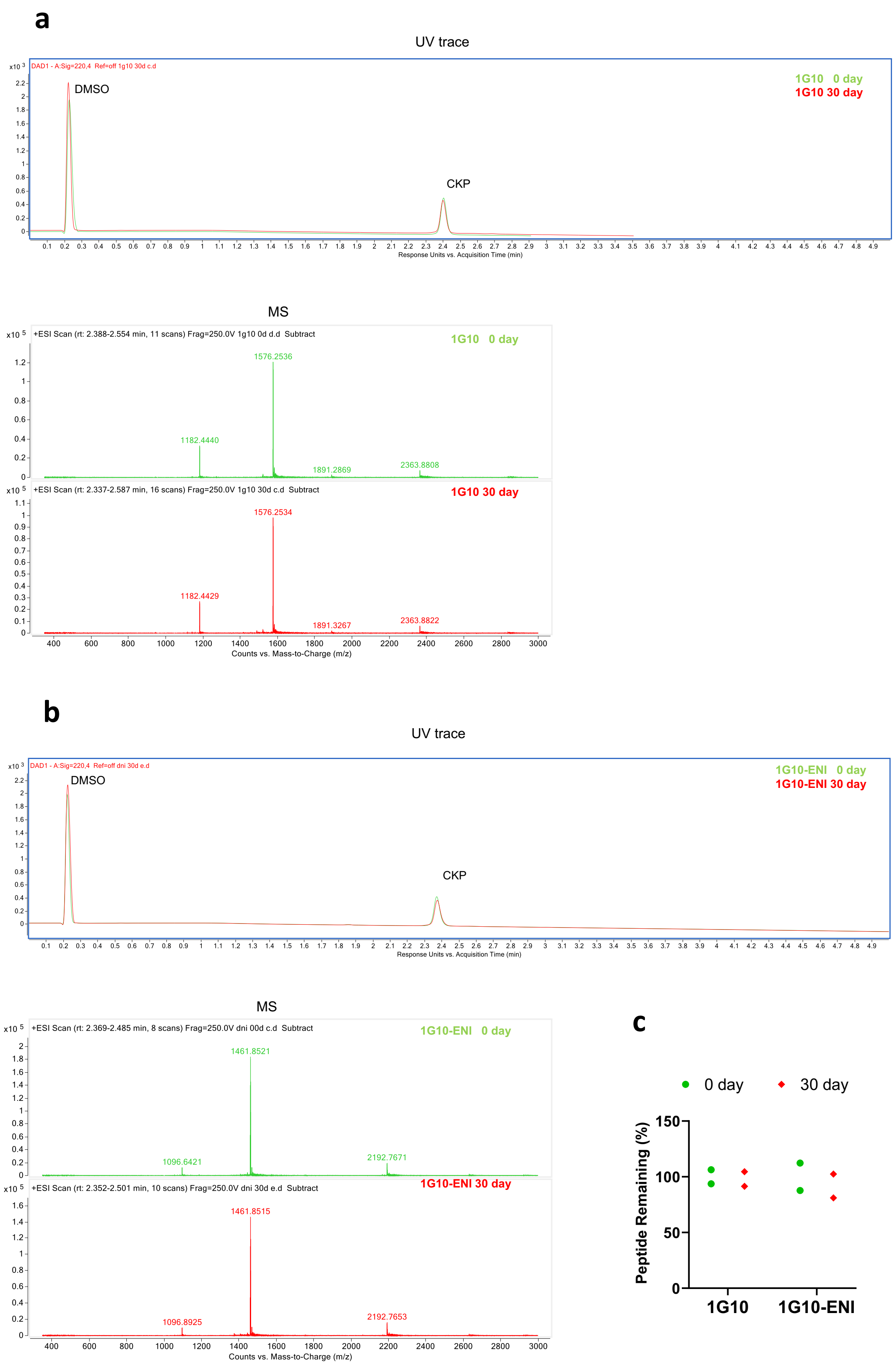
a
UV trace
DAD1 - A:Sig=220,4 Ref=off 1g10 30d c.d
1G10 0 day
1G10 30 day
DMSO
CKP
Response Units vs. Acquisition Time (min)
MS
+ESI Scan (rt: 2.388-2.554 min, 11 scans) Frag=250.0V 1g10 0d d.d Subtract
1G10 0 day
1576.2536
1182.4440
1891.2869
2363.8808
+ESI Scan (rt: 2.337-2.587 min, 16 scans) Frag=250.0V 1g10 30d c.d Subtract
1G10 30 day
1576.2534
1182.4429
1891.3267
2363.8822
Counts vs. Mass-to-Charge (m/z)
b
UV trace
DAD1 - A:Sig=220,4 Ref=off dni 30d e.d
1G10-ENI 0 day
1G10-ENI 30 day
DMSO
CKP
Response Units vs. Acquisition Time (min)
MS
+ESI Scan (rt: 2.369-2.485 min, 8 scans) Frag=250.0V dni 00d c.d Subtract
1G10-ENI 0 day
1461.8521
1096.6421
2192.7671
+ESI Scan (rt: 2.352-2.501 min, 10 scans) Frag=250.0V dni 30d e.d Subtract
1G10-ENI 30 day
1461.8515
1096.8925
2192.7653
Counts vs. Mass-to-Charge (m/z)
c
0 day
30 day
Peptide Remaining (%)
1G10
1G10-ENI

**Supplementary Figure 13. Stability of the potent HTRA1 inhibitors 1G10 and 1G10-ENI. a. and b.** Representative UV trace (220 nm) and MS analysis of 1G10 (a) and 1G10-ENI (b) at 0 day (green) and 30 days (red) after incubation at 37° C at a concentration of 0.3 mM (~ 1 mg/ml) in PBS containing 0.15% DMSO. There was no change in peptide mass at 30 days, indicating that the peptides did not undergo any fragmentation or chemical modification, but remained intact. **c.** Representative data of two independent experiments showing quantification of intact peptide remaining after 30 days of exposure. The values obtained from multiple HPLC runs were averaged and the amounts of peptide at day 0 (green round dots) and after 30 days (red diamond dots) were shown.

## Supplementary Tables

### Supplementary Table 1: Binding kinetics of affinity-improved CKPs to HTRA1$^{PD(SA)}$ as determined by SPR

| CKP | $k_{on}$ [$M^{-1}$ $s^{-1}$] x$10^4$ | $k_{off}$ [s-1] x$10^{-4}$ | $K_D$ [nM] |
|---|---|---|---|
| *Initial library screen* | | | |
| 3B3 | 1.1 ± 0.2 | 3.8 ± 0.5 | 34.0 ± 3.5 |
| 3A7 | 4.5 ± 0.3 | 2.8 ± 0.2 | 6.2 ± 0.2 |
| *Affinity maturation* | | | |
| 1A8 | 8.2 ± 3.5 | 0.5 ± 0.1 | 0.66 ± 0.42 |
| 2D5 | 10.5 ± 0.9 | 1.0 ± 0.1 | 0.94 ± 0.12 |
| 1A7 | 9.4 ± 1.3 | 6.5 ± 0.7 | 7.0 ± 0.5 |
| 1C10 | 7.7 ± 2.4 | 4.7 ± 0.1 | 6.5 ± 2.2 |
| CPI* | -- | -- | No binding |
| *Combinations* | | | |
| 1D3-ENI | 29.1 ± 7.6 | 1.0 ± 0.1 | 0.38 ± 0.18 |
| 1G10-ENI | 28.5 ± 2.5 | 1.2 ± 0.3 | 0.44 ± 0.14 |

*No binding detected up to 10 µM CPI (carboxypeptidase inhibitor)
Values indicated in the table are the mean ± S.D. of three or more independent experiments.
Source data are available as a Source Data File

## Supplementary Table 2. Data collection and refinement statistics

| | 3B3:HTRA1$^{PD(SA)}$ | 3A7:HTRA1$^{PD(SA)}$ | 1A8:HTRA1$^{PD(SA)}$ | 1G10:HTRA1$^{PD(SA)}$ |
|---|---|---|---|---|
| **Data collection** | | | | |
| Space group | $P2_12_12_1$ | $P2_12_12_1$ | $P2_12_12_1$ | $P2_12_12_1$ |
| Cell dimensions | | | | |
| $a, b, c$ (Å) | 47.69, 84.61, 200.80 | 48.31, 86.81, 199.71 | 116.0, 152.7, 165.8 | 120.82, 155.36, 173.40 |
| α, β, γ (°) | 90, 90, 90 | 90, 90, 90 | 90, 90, 90 | 90, 90, 90 |
| Resolution (Å) | 50.45-3.05 (3.16-3.05)* | 46.92-2.87 (2.98-2.87)* | 41.46-2.97 (3.07-2.97)* | 95.38-3.18 (3.30-3.18)* |
| $R_{merge}$ | 0.124 (0.948) | 0.096 (1.210) | 0.129 (1.270) | 0.046 (0.943) |
| $I / \sigma I$ | 9.0 (2.0) | 13.0 (1.6) | 12.9 (1.4) | 16.2 (1.5) |
| Completeness** | | | | |
| % (spherical) | 45.9 (7.8) | 57.6 (9.4) | 77.7 (16.4) | 83.4 (22.7) |
| % (ellipsoidal) | 83.3 (63.8) | 90.5 (64.1) | 94.8 (62.6) | 93.1 (51.7) |
| Redundancy | 6.2 (5.0) | 6.3 (5.7) | 6.2 (6.2) | 4.7 (4.8) |
| **Refinement** | | | | |
| Resolution (Å) | 50.54-3.05 | 46.92 - 2.87 | 41.46 -2.97 | 20.0 - 3.18 |
| No. reflections | 7422 (50) | 11456 (34) | 47914 (195) | 46170 (484) |
| $R_{work}$ / $R_{free}$ | 0.260/ 0.295 | 0.274/ 0.293 | 0.270/ 0.296 | 0.259/ 0.282 |
| No. atoms | | | | |
| Protein | 4705 | 4826 | 19018 | 18760 |
| Ligand/ion | - | 5 | - | 34 |
| Water | - | - | 7 | 5 |
| $B$-factors | | | | |
| Protein | 81.07 | 78.76 | 87.18 | 138.77 |
| Ligand/ion | - | 91.03 | - | 149.16 |
| Water | - | - | 64.49 | 105.05 |
| R.m.s. deviations | | | | |
| Bond lengths (Å) | 0.003 | 0.003 | 0.002 | 0.002 |
| Bond angles (°) | 0.75 | 0.58 | 0.52 | 0.49 |
| PDB code | 8SDM | 8SDP | 8SE7 | 8SE8 |

*Values in parentheses are for highest-resolution shell
**Completeness after anisotropic correction with STARANISO

## References:

1. Chiche L, Gaboriaud C, Heitz A, Mornon JP, Castro B, Kollman PA. Use of restrained molecular dynamics in water to determine three-dimensional protein structure: prediction of the three-dimensional structure of Ecballium elaterium trypsin inhibitor II. *Proteins* **6**, 405-417 (1989).

2. Rees DC, Lipscomb WN. Refined crystal structure of the potato inhibitor complex of carboxypeptidase A at 2.5 A resolution. *J Mol Biol* **160**, 475-498 (1982).

3. Kasperkiewicz P, Poreba M, Snipas SJ, Lin SJ, Kirchhofer D, Salvesen GS, Drag M. Design of a Selective Substrate and Activity Based Probe for Human Neutrophil Serine Protease 4. *PLoS One* **10**, e0132818 (2015).
