# Supplementary material for: Cystine-knot peptide inhibitors of HTRA1 bind to a cryptic pocket within the active site region
Source: Nat Commun. 2024 May 22;15:4359. doi: 10.1038/s41467-024-48655-w (PMC11111691; doi:10.1038/s41467-024-48655-w)
Supplement: Supplementary file 3 — Reporting Summary [file 41467_2024_48655_MOESM3_ESM.pdf]

nature portfolio

Corresponding author(s): Daniel Kirchhofer

Last updated by author(s): Apr 26, 2024

Reporting Summary

Nature Portfolio wishes to improve the reproducibility of the work that we publish. This form provides structure for consistency and transparency in reporting. For further information on Nature Portfolio policies, see our Editorial Policies and the Editorial Policy Checklist.

Statistics

For all statistical analyses, confirm that the following items are present in the figure legend, table legend, main text, or Methods section.

n/a	Confirmed	
☐	☒	The exact sample size (*n*) for each experimental group/condition, given as a discrete number and unit of measurement
☐	☒	A statement on whether measurements were taken from distinct samples or whether the same sample was measured repeatedly
☐	☒	The statistical test(s) used AND whether they are one- or two-sided<br>*Only common tests should be described solely by name; describe more complex techniques in the Methods section.*
☒	☐	A description of all covariates tested
☒	☐	A description of any assumptions or corrections, such as tests of normality and adjustment for multiple comparisons
☐	☒	A full description of the statistical parameters including central tendency (e.g. means) or other basic estimates (e.g. regression coefficient) AND variation (e.g. standard deviation) or associated estimates of uncertainty (e.g. confidence intervals)
☐	☒	For null hypothesis testing, the test statistic (e.g. *F*, *t*, *r*) with confidence intervals, effect sizes, degrees of freedom and *P* value noted<br>*Give P values as exact values whenever suitable.*
☒	☐	For Bayesian analysis, information on the choice of priors and Markov chain Monte Carlo settings
☒	☐	For hierarchical and complex designs, identification of the appropriate level for tests and full reporting of outcomes
☒	☐	Estimates of effect sizes (e.g. Cohen's *d*, Pearson's *r*), indicating how they were calculated

Our web collection on statistics for biologists contains articles on many of the points above.

Software and code

Policy information about availability of computer code

Data collection	For X-ray data collection, refinement and graphics we used the following softwares: iMosflm, STARANISO, Phaser, Phenix, Coot, XDS, PyMOL.
Data analysis	Structural data was analyzed and visualized using Pymol version 2.5.2.<br>SPR data were analyzed by using the GE Biacore S200 instrument and analyzed by Biacore S200 evaluation software version 1.0.<br>Enzymatic data was analyzed with Graphpad Prism 10.<br>SDS-PAGE and fluorescent gels were analyzed using Gel Doc Imager Software (Biorad).

For manuscripts utilizing custom algorithms or software that are central to the research but not yet described in published literature, software must be made available to editors and reviewers. We strongly encourage code deposition in a community repository (e.g. GitHub). See the Nature Portfolio guidelines for submitting code & software for further information.

Data

Policy information about availability of data

All manuscripts must include a data availability statement. This statement should provide the following information, where applicable:

- Accession codes, unique identifiers, or web links for publicly available datasets
- A description of any restrictions on data availability
- For clinical datasets or third party data, please ensure that the statement adheres to our policy

The data that support this study are available from the corresponding authors upon request. The X-ray structures of the complexes of 3B3:HTRA1PD(SA) (PDB:

8SDM), 3A7:HTRA1PD(SA) (PDB: 8SDP), 1A8:HTRA1PD(SA) (PDB: 8SE7) and 1G10:HTRA1PD(SA) (PDB: 8SE8) have been submitted to the Protein Data Bank (https://www.rcsb.org/). All reagents are available from the lead contact under a material transfer agreement with Genentech. Source data underlying Figs. 1, 2, 4, 6, Supplementary Figures. 2, 3, 6, 7, 8, 10, 12, 13 and Supplementary Table 1 are provided. Source data are provided as a Source Data file.

Research involving human participants, their data, or biological material

Policy information about studies with human participants or human data. See also policy information about sex, gender (identity/presentation), and sexual orientation and race, ethnicity and racism.

Reporting on sex and gender	The C32 cell line (ATCC #CRL-1585) was derived from the skin of a 53-year-old Caucasian male with amelanotic melanoma
Reporting on race, ethnicity, or other socially relevant groupings	The C32 cell line (ATCC #CRL-1585) was derived from the skin of a 53-year-old Caucasian male with amelanotic melanoma
Population characteristics	Not applicable
Recruitment	Not applicable
Ethics oversight	Not applicable

Note that full information on the approval of the study protocol must also be provided in the manuscript.

Field-specific reporting

Please select the one below that is the best fit for your research. If you are not sure, read the appropriate sections before making your selection.

☒ Life sciences ☐ Behavioural & social sciences ☐ Ecological, evolutionary & environmental sciences

For a reference copy of the document with all sections, see nature.com/documents/nr-reporting-summary-flat.pdf

Life sciences study design

All studies must disclose on these points even when the disclosure is negative.

Sample size	This study characterizes in vitro protein activity and inhibitor binding affinity. All biological replicates were examined and we did not sample only part of them. Therefore, sample size was not predetermined for this study.
Data exclusions	No data was excluded.
Replication	SPR experiments for determining KD values were carried out at least three times on different experimental days using freshly made reagents and newly made dilution series of analytes. All attempts at replication were successful.<br>Enzymatic assays were carried out at least three times on different experimental days using freshly made reagents. All attempts at replication were successful.<br>SDS-PAGE gels and fluorescent gels were repeated at least 3 times and representative images are shown in the paper. Appropriate controls were run in parallel. All attempts at replication were successful.
Randomization	Our experiments were not randomized. This statistical consideration is not relevant to our study because of the nature of biochemical, structural experiments performed in the work.
Blinding	The investigators were not blinded. Blinding is not technically or practically feasible for the experiments in this work.

Reporting for specific materials, systems and methods

We require information from authors about some types of materials, experimental systems and methods used in many studies. Here, indicate whether each material, system or method listed is relevant to your study. If you are not sure if a list item applies to your research, read the appropriate section before selecting a response.

Materials & experimental systems

n/a	Involved in the study	
☐	☒	Antibodies
☐	☒	Eukaryotic cell lines
☒	☐	Palaeontology and archaeology
☒	☐	Animals and other organisms
☒	☐	Clinical data
☒	☐	Dual use research of concern
☒	☐	Plants

Methods

n/a	Involved in the study	
☒	☐	ChIP-seq
☒	☐	Flow cytometry
☒	☐	MRI-based neuroimaging

Antibodies

Antibodies used

IgG94. anti-HtrA1 antibody developed in house (Biochem J. 2015 Dec 1;472(2):169-81) and used in 0.5 µM in this study.
Anti-M13 antiboy-HRP (Creative Diagnostics, cat# CAB-655M) was used in 1:10000 dilution.

Validation

IgG94 has been validated to inhibit recombinant HtrA1 and HtrA1 from human cancer cell line C32 at 0.5 µM and IgG94 binding to HtrA1 has been resolved in cryoEM structural study (Biochem J. 2015 Dec 1;472(2):169-81)
Anti-M13 antiboy-HRP have been validated for multiple applications (WB, ELISA, FC, etc.) by the vendor.

Eukaryotic cell lines

Policy information about cell lines and Sex and Gender in Research

Cell line source(s)

The C32 cell line (ATCC #CRL-1585) was derived from the skin of a 53-year-old Caucasian male with amelanotic melanoma

Authentication

Authentication by STR analysis. see Source Data file for details.

Mycoplasma contamination

The C32 cell line was not tested for Mycoplasma contamination

Commonly misidentified lines
(See ICLAC register)

Not applicable

Plants

Seed stocks

Not applicable

Novel plant genotypes

Not applicable

Authentication

Not applicable
